# Micro-scale modelling of the urban wind speed for air pollution applications

**DOI:** 10.1038/s41598-019-50033-2

**Published:** 2019-10-03

**Authors:** Thor-Bjørn Ottosen, Matthias Ketzel, Henrik Skov, Ole Hertel, Jørgen Brandt, Konstantinos E. Kakosimos

**Affiliations:** 1 Institute of Science and the Environment, University of Worcester, Worcester, UK; 2 Department of Chemical Engineering, Biotechnology and Environmental Technology, University of Southern Denmark, Odense, Denmark; 3 Department of Chemical Engineering, Texas A&M University at Qatar, Doha, Qatar; 4 Department of Environmental Science, Aarhus University, Roskilde, Denmark; 5 Global Centre for Clean Air Research (GCARE), University of Surrey, Guildford, GU2 7XH United Kingdom

**Keywords:** Environmental sciences, Atmospheric dynamics, Engineering

## Abstract

Modelling wind speeds in urban areas have many applications e.g. in relation to assessment of wind energy, modelling air pollution, and building design and engineering. Models for extrapolating the urban wind speed exist, but little attention has been paid to the influence of the upwind terrain and the foundations for the extrapolation schemes. To analyse the influence of the upwind terrain and the foundations for the extrapolation of the urban wind speed, measurements from six urban and non-urban stations were explored, and a model for the urban wind speed with and without upwind influence was developed and validated. The agreement between the wind directions at the stations is found to be good, and the influence of atmospheric stability, horizontal temperature gradients, land-sea breeze, temperature, global radiation and Monin-Obukhov Length is found to be small, although future work should explore if this is valid for other urban areas. Moreover, the model is found to perform reasonably well, but the upwind influence is overestimated. Areas of model improvement are thus identified. The upwind terrain thus influences the modelling of the urban wind speed to a large extent, and the fundamental assumptions for the extrapolation scheme are fulfilled for this specific case.

## Introduction

The urban wind speed has been an area of research, as part of urban climatology studies, since the 1930s^1^. The initial research interest focused on large-scale effects^2,3^ and was subsequently spurred by the interest in the problems of air pollution^4,5^. The topic of urban wind speed for air pollution applications has remained an active area of research until today^6–11^. In recent years, the urban wind speed has as well been of interest for urban wind energy (e.g.^12–14^) or assessments of wind loadings on tall buildings^15^.

Often, financial and technical constraints prevent the use of measurements to assess the urban wind speed. Models of the urban wind speed are therefore often used to estimate the urban wind speed at many locations. The present study is motivated by the use of the modelled urban wind speed as input to regulatory air pollution models, since previous work^16,17^ have shown the importance of this input. These models are characterised by being fast, generally applicable, reliable, and simple to use^18–20^. The same principles continue to guide the development of such models even today despite the evolution of high performance computing facilities. This is the case, since the needs have evolved and increased as well, demanding longer and finer time scales and more and diverse receptor locations. In line with this approach, focus of the present study is on modelling hourly averaged wind speeds in the roughness sublayer (using the terminology of^21^), since this is the averaging time and relevant height used in regulatory air quality models.

The urban wind speed can be modelled using different approaches:

1. Large-scale numerical weather prediction models (e.g.^22,23^).
2. Physical models (e.g. wind or water tunnels) (e.g.^24^).
3. Computational fluid dynamics (CFD) models (e.g.^25,26^).
4. Models based on analytical horizontal and vertical extrapolations (e.g. the references in Table 4 in the Supplementary Material).

The design principles behind regulatory air quality models effectively limits the models in the present context to models applying analytical horizontal and vertical extrapolations.

A number of recent models of the urban wind speed using analytical horizontal and vertical extrapolations have been presented in the literature^7,12,13,15,27–30^. An overview of recent models of the urban wind speed using analytical horizontal and vertical extrapolations are given in Table 4 in the Supplementary Material. All studies are using either input from a nearby airport or various wind speed databases as input. The extrapolation method termed *Profile* in Table 4 is using a double vertical-horizontal interpolation following the approach of^31^. The studies in Table 4 are using logarithmic profiles, whose application and validity are discussed in more detail in the Supplementary Material. The extrapolation method termed *IBL* (Internal Boundary Layer) is using an extrapolation approach across multiple internal boundary layers following either^32^ and^33^ or^34^. Apart from the studies in Table 4^35^, fitted straight lines to scatter plots of measured rural and urban wind speed data from Birmingham, UK (2 × 4 weeks); Copenhagen, Denmark (1 year); Lisbon, Portugal (3 months); and Barcelona, Spain (1 year). Their study was part of European Cooperation in Science and Technology (COST) 715^6^ and concluded that the relationship was site specific and required more investigation. The many different approaches presented in Table 4 highlights the need for model comparisons; however, only a few model comparisons are available in the literature^36^, and given the vast number of approaches a comparison is beyond the scope of the present study.

Previous work on the influence of the upstream terrain on urban wind speed measurements was reviewed, and a variation of a factor of two in the drag coefficient for approximately 20° wind direction change was shown^37^. This development in measurements has, to the best of the authors’ knowledge, not been reflected in the modelling. The influence of the upwind terrain above the blending height has been modelled^13^, and the upwind influence as a function of four wind directions (North, South, East, and West) has been modelled as well^28^. As seen from Table 4 in the Supplementary Material, neither of these studies have performed validation on the hourly time scale, the time scale of relevance for the present study, and the accuracy of these modelling approaches, for this time scale, is thus unknown. Moreover, the studies in Table 4 in the Supplementary Material have furthermore paid less attention to whether the fundamental assumptions for the extrapolation schemes are fulfilled. It is thus an open question, to what extent the upwind terrain influences modelling of the urban roughness sublayer wind speed, and to what extent, the assumptions for the extrapolation technique are fulfilled.

To asses the validity of the assumptions for the extrapolation technique, exploratory data analyses of meteorological data were performed. To assess the influence of the upwind terrain in models of the urban wind speed, a new model for the urban wind speed, with (henceforth referred to as the *advanced model*) and without (henceforth referred to as the *simple model*) upwind influence, was developed and validated using the measurements. Given the challenge of this task, with respect to both modelling and measurements, the present model should be considered a first step in the direction of a more generally applicable urban wind speed model.

## Results

### Analysis of fundamental model assumptions

#### Influence of wind speed and direction

**Figure 1:**
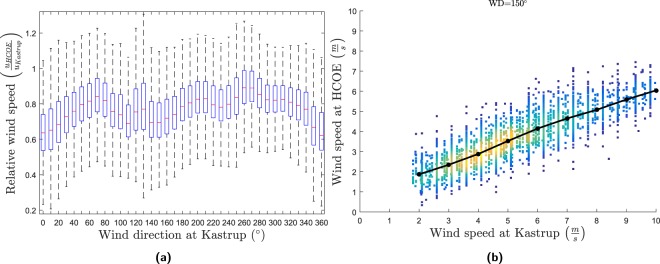
Boxplot of the relative wind speed between HCOE (urban mast) and Kastrup (airport) (**1a**) and scatterplot of the wind speed at HCOE versus the wind speed at Kastrup for a specific wind direction in Kastrup of 150° (**1b**). The red lines in (**a**) are the medians, the boxes are the 25 and 75 percentiles, and the whiskers are ≈2.7*σ*. The colors in (**b**) represent the relative density of points. Wind speeds below 1.75 m s^−1^ and above 10 m s^−1^ in Kastrup have been removed.

The wind speed extrapolation model uses only the wind direction and wind speed as input data. That the relative wind speed is wind direction dependent is evident from the box plot in Fig. 1. Whereas the median value is changing with wind direction, the scatter in the relative wind speed appears to be independent of wind direction. As an example, the wind speed at HCOE is plotted against the wind speed in Kastrup for an arbitrary wind direction of 150° in Fig. 1. It is evident from the figure that the relative wind speed is wind speed dependent. It can thus be confirmed from Fig. 1 that the extrapolated wind is dependent on wind speed and wind direction.

It is moreover evident from Fig. 1 that the wind speed at the HCOE (urban background station at the H. C. Oersteds Institute, University of Copenhagen. See Supplementary Material for station characteristics) is not constant for a given input wind speed and a given wind direction. Instead, for each input wind speed and direction, there is a distribution of wind speeds at HCOE. This distribution in wind speeds is caused by turbulence in the atmosphere at all length scales between the two stations. Since this variance is not modelled in the present application, the model validation will be divided into two parts: To assess the performance of the model, it is validated against the median values for each wind speed and wind direction. The median is chosen instead of the mean since the distributions are not always symmetric. To explore the predictability of the hourly wind speed, the model is compared to the full dataset.

#### Influence of horizontal temperature gradient and other effects

Horizontal temperature gradients will cause poor performance of this scheme, since this will cause a change in pressure gradient with height^31^. The influence of horizontal temperature gradient was examined by analysing the relative wind speed for the Copenhagen and Aarhus airport-urban mast station pairs as a function of temperature difference between the two stations. The analysis showed that the mean temperature in Aarhus is 0.88 °C higher than the airport with a standard deviation of 1.53 °C. For Copenhagen the mean and standard deviation is respectively 0.33 °C and 1.15 °C. These are small temperature differences between the stations which also explains why the analysis showed almost no effect of horizontal temperature gradient.

The presence of land-sea breezes was examined by plotting the diurnal variation in the wind direction at Kastrup Airport. The station was found not to be influenced by land-sea breeze.

In an attempt to explain the remaining variance in the measurements, scatter plots of the remaining variance were produced and these showed only vague connections to the other meteorological variables measured (being temperature and global radiation)^38^. No connection was likewise found between the remaining variance and the atmospheric stability represented by the Monin-Obukhov Length (the height at which the production of turbulence by mechanical and buoyancy forces is equal^39^, [p. 747]) calculated based on meteorological data from Meteorological Model 5 (air density, temperature, friction velocity and surface heat flux) on a 5.6 km × 5.6 km resolution for the year 2012. This indicates that wind speed and wind direction are the determining variables among the inputs available.

The analysis of the agreement in wind direction between the stations and the influence of atmospheric stability can be found in the Supplementary Material.

The above analyses show that the present case study is dominated by mesoscale weather patterns, which allows the use of the presented extrapolation scheme. Other cities have reported temperature gradients up to 8 °C^40^, and mixed results have been reported as to the influence of atmospheric stability in urban areas^37,41–43^. In these cases, a more advanced modelling approach might be required.

#### Analysis of the spectral representation of data

**Figure 2:**
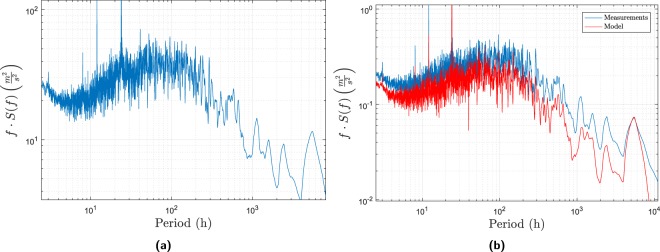
Lomb-Scargle periodogram for the covariance of the wind speed between Kasptup and HCOE (**1a**) and the variance of the measured and modelled wind speed at HCOE (**1b**).

To analyse the properties of the different ways to represent wind speed data for two stations (absolute wind speeds, relative wind speeds, or wind speed differences), the spectral representation was analysed. In this section, the term *model* is used to mean the *advanced model*, since the *simple model* will have the same spectra as the measurements. Since HCOE is the longest time series in the present study, the periodogram of the covariance between the wind speed at this station and the wind speed in Kastrup is shown in Fig. 2. It is evident that the two stations have high covariance at the frequencies corresponding to the diurnal variation plus the harmonics of this frequency. Moreover, there is an annual cycle seen in the wind speed. Naturally, these wind speed cycles will influence the stations in the same way owing to the nearness of the stations to each other. It can also be seen that the covariance periodogram is dominated by frequencies in the range ≈20 h to ≈130 h. This is the time scale for changes in the weather pattern on the mesoscale. This is also seen in Fig. 2 where it is evident that both the model and the measurements have the same spectrum.

**Figure 3:**
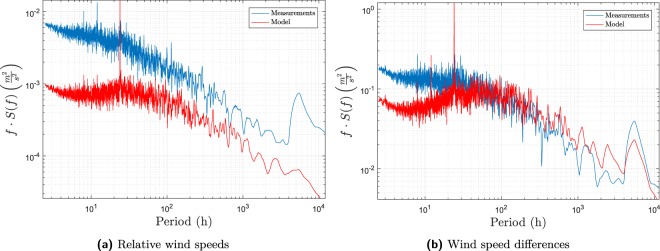
Lomb-Scargle periodogram of the variance of the relative wind speed (**3a**) and the wind speed differences (**3b**) between Kastrup and HCOE for both the model and the measurements.

The periodogram of the relative wind speed is shown in Fig. 3. The peaks for the diurnal and annual variation can also be found in this spectrum. The *y*-axes in Fig. 2 are different since the total variance of the relative wind speed is much smaller than the total covariance of the two wind speeds, as shown in Fig. 2. However, it is shown from this figure that the mesoscale effects have been diminished, as seen from the absence of high values in the 10 h to 100 h range. The local scale phenomena are now dominating the variance in the representation, which is desirable as this is the focus of the present model. Unfortunately, this pattern is not reproduced in the model output, which invalidates this representation for model validation. The same phenomenon is seen for the wind speed difference in Fig. 3. The explanation is that the absolute wind speeds are used as input to the model. Since these depend heavily on mesoscale effects, the mesoscale effects will also be present in the output. The representation where the model output and the measurements have the same spectrum is therefore the absolute wind speeds. This representation is therefore used in the model validation.

Figure 2 showed that the wind speed data is not identically distributed due to the periodicities in data, and since data are strongly autocorrelated, the wind speed data is not independent. To the best of the authors knowledge, there is no standard way to transform wind speed data into an independent and identically distributed dataset. Since independent and identically distributed data is a fundamental assumption for several statistical analyses (e.g. correlation, analysis of variance (ANOVA), etc.) this effectively limits the ways the data can be analysed.

### Model validation

#### Validation against median values

**Figure 4:**
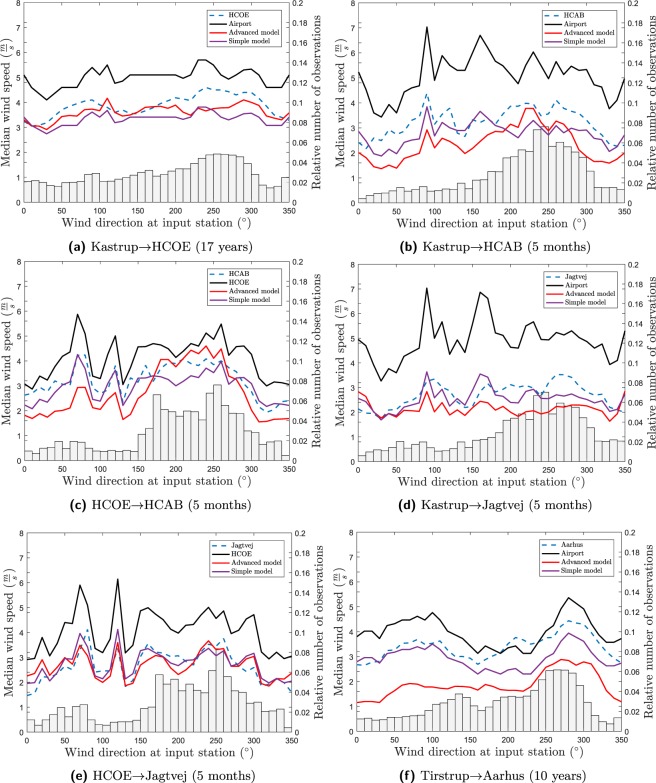
Plot of the median wind speed for the measurements (blue, dashed), the advanced model (red), the simple model (purple,) and the input wind speed (black) (either the closest mast or airport station) as a function of wind direction for the three stations in Copenhagen using the two available input stations plus Aarhus using Tirstrup Airport as input. The bar charts represent the relative number of observations for each wind direction and are plotted on the right y-axis.

The model output and the measurements as a function of wind direction can be seen in Fig. 4. As seen from Fig. 4, both models predict the large wind speed reduction from the airport to the city and the smaller wind speed reduction from the mast to the roof. The curves for the median input wind speed are not identical since each one cover different periods in time for respectively the permanent stations and the campaign stations. The figures also show that both models tend to underestimate the wind speed, more so for the advanced model than the simple model.

For Kastrup→HCOE the advanced model reproduces the wind direction dependency with good performance. HCOE is, as seen in the maps in the attached Google maps file, influenced by a large park for easterly winds and subsequently influenced by a large high rise building at 140° to 150°; these features are nicely reproduced by the advanced model and not seen in the simple model. This pattern is, to some extent, also seen for HCOE→Jagtvej (urban roof level station at Jagtvej, Copenhagen. See Supplementary Material for station characteristics) and HCOE→HCAB (urban roof level station at H. C. Andersens Boulevard, Copenhagen. See Supplementary Material for station characteristics) and in Kastrup→Jagtvej due to the short distance between HCOE and Jagtvej. Both the advanced model and the simple model are underestimating the wind speed for westerly wind directions, more so for the simple model than the advanced model for this station. This can potentially be connected to some areas of detached houses upstream of the Kastrup station whose influence is underestimated in both models. This is emphasised by the fact that all models underestimate westerly wind speeds when Kastrup is used as input.

HCAB is influenced by an amusement park with correspondingly low roughness for south-westerly wind directions thus giving a characteristic wind direction pattern. This pattern is overestimated in the advanced model for HCOE→HCAB and not present in the simple model. The underestimation for Kastrup→Jagtvej for south-westerly winds can potentially be linked to something (probably the aforementioned detached houses upwind of the Kastrup station) not accurately modelled in Kastrup. This is the case since the wind direction pattern is nicely reproduced by the model when HCOE is used as input. HCOE→Jagtvej is nicely reproduced by both models. This means that this represents a particularly simple situation where a simpler approach can be useful. The reason is the short distance between the stations meaning that the wind speed and direction will be extremely identical for the two stations.

Tirstrup→Aarhus (urban background station at the Aarhus town hall, Aarhus. See Supplementary Material for station characteristics) is a special case since the airport wind speed is only slightly larger than the urban wind speed. For a small interval around 200° the urban wind speed is actually larger than the airport wind speed. This is the case since the urban station is only a few meters higher than the airport station. Moreover, the airport station is influenced by forest areas close to the station. Nevertheless, the model underestimation for the advanced model is more pronounced for this station compared with the other stations. For the interval $[0^\circ :150^\circ ]$ the simple model is reproducing observations fairly well whereas the forest areas for southern wind directions are underestimated in both models, more so for the advanced model than the simple model.

The comparison between the simple model and the advanced model indicates that the upwind influence is overestimated in the advanced model for some wind direction intervals. This can be seen in e.g. Kastrup→HCAB $[300^\circ :350^\circ ]$ and HCOE→HCAB $[50^\circ :150^\circ ]$. It is characteristic that these wind direction intervals are having few observations as seen in the bar charts in Fig. 4. The effect of this is that the curve is disproportionately influenced by individual weather events creating e.g. the spikes in the curves for both Jagtvej and HCAB. It is likely that the wind direction plot for the urban mast stations will change as the time series expand and the spikes become smoothed out. It is therefore too early to conclude on the upwind influence in the model for these stations. The effect is also pronounced for Aarhus where the simple model is almost perfect for $[0^\circ :150^\circ ]$. Future work should aim at reducing the upwind influence for this station. On the other hand, it is shown that the simple approach is insufficient for stations with inhomogeneous upwind roughness such as Kastrup, HCOE, and HCAB.

A similar pattern can be seen in Table 1. In general both models have smaller relative deviations (except the advanced model for Aarhus) compared with the input. The simple model has in general a smaller relative deviation indicating the previously described problems with the upwind influence in the model. On the other hand, the advanced model generally have a higher correlation coefficient indicating a stronger association between the results from the advanced model and the measurements. It can be seen that the correlation is dropping for Jagtvej. It has to be noted that the correlation between HCOE and Jagtvej is already quite high, and it is thus a challenge to improve on this figure. This shows a need for a more advanced model for longer extrapolation distances. For Kastrup→Jagtvej the low correlation is explained by the urban station being, as seen from the attached Google Maps file, located in quasi-homogeneous surroundings also leading to a lower variance for this station. This means that the correlation coefficient is a skewed performance measure for this combination of stations.

**Table 1:** Maximum, minimum, and mean absolute relative deviation among the median values as a function of wind direction of the input, and the two models.

Receptor	Model	Max (%)	Min (%)	Mean (%)	Correlation coefficient (*R*^2^)
HCOE	Kastrup	57	11	30	0.67
HCOE	Advanced model	20	0	8	0.74
HCOE	Simple model	26	1	14	
HCAB	Kastrup	116	23	61	0.63
HCAB	Advanced model	49	4	27	0.80
HCAB	Simple model	33	0	17	
Jagtvej	Kastrup	165	46	86	0.58
Jagtvej	Advanced model	43	2	24	0.12
Jagtvej	Simple model	37	1	13	
HCAB	HCOE	55	6	29	0.85
HCAB	Advanced model	44	1	22	0.84
HCAB	Simple model	23	1	11	
Jagtvej	HCOE	99	24	57	0.85
Jagtvej	Advanced model	54	0	14	0.76
Jagtvej	Simple model	33	0	12	
Aarhus	Tirstrup	52	0	21	0.64
Aarhus	Advanced model	59	28	46	0.84
Aarhus	Simple model	37	0	14	

The simple model has the same correlation as the input and is therefore not shown.

**Figure 5:**
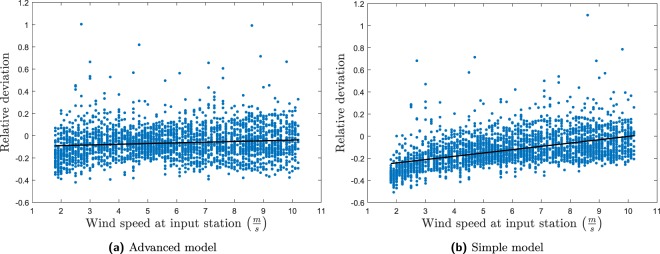
Plot of the relative deviation between the model and the measurements as a function of input wind speed. The black line is a linear trend line.

To analyse the dependency on wind speed, the relative deviation $(\tfrac{{u}_{{\rm{model}}}-{u}_{{\rm{measurements}}}}{{u}_{{\rm{measurements}}}})$ was calculated for each wind speed and wind direction. A linear trend was subsequently fitted to these data. A perfect model will have a slope and intercept of zero. An example of this for Kastrup→HCOE is shown in Fig. 5. The slope and intercept for the trend line for all the streets for both models can be found in Table 2. From Table 1 the advanced model appeared to be somewhat better than the simple model for Kastrup→HCOE. From Fig. 5 it is evident that the median performance of the simple model covers some significant differences between high and low wind speeds, with the bias on the low wind speeds being up to 30%. This is not seen for the advanced model, where the bias is fairly homogeneously distributed with respect to wind speed.

**Table 2:** Slope and intercept of a linear function fitted to the relative deviation between model and measurements as a function of input wind speed.

Receptor	Model	Input	Slope (·10^−2^)	Intercept
HCOE	Advanced model	Kastrup	0.62	−0.10
HCOE	Simple model	Kastrup	2.99	−0.30
HCAB	Advanced model	Kastrup	1.97	−0.34
HCAB	Simple model	Kastrup	3.94	−0.33
Jagtvej	Advanced model	Kastrup	0.38	−0.20
Jagtvej	Simple model	Kastrup	2.95	−0.17
HCAB	Advanced model	HCOE	2.85	−0.24
HCAB	Simple model	HCOE	0.94	−0.10
Jagtvej	Advanced model	HCOE	−0.15	0.01
Jagtvej	Simple model	HCOE	0.16	0.02
Aarhus	Advanced model	Tirstrup	1.21	−0.52
Aarhus	Simple model	Tirstrup	4.75	−0.33

A similar trend can be seen for the slope and intercept of the trend line for the extrapolations in Table 2. This indicates that using the modelled urban boundary layer height for horizontal extrapolation performs better than assuming a constant extrapolation height of 4*h*_*r*_. For HCOE→Jagtvej the performance of the two models is approximately equal whereas for HCOE→HCAB the simple model performs better. This can be explained by the large low-roughness areas (large parks/amusement parks) close to these two stations influencing u_*_. Future work should aim at reducing this effect in the advanced model.

#### Validation against the full dataset

**Figure 6:**
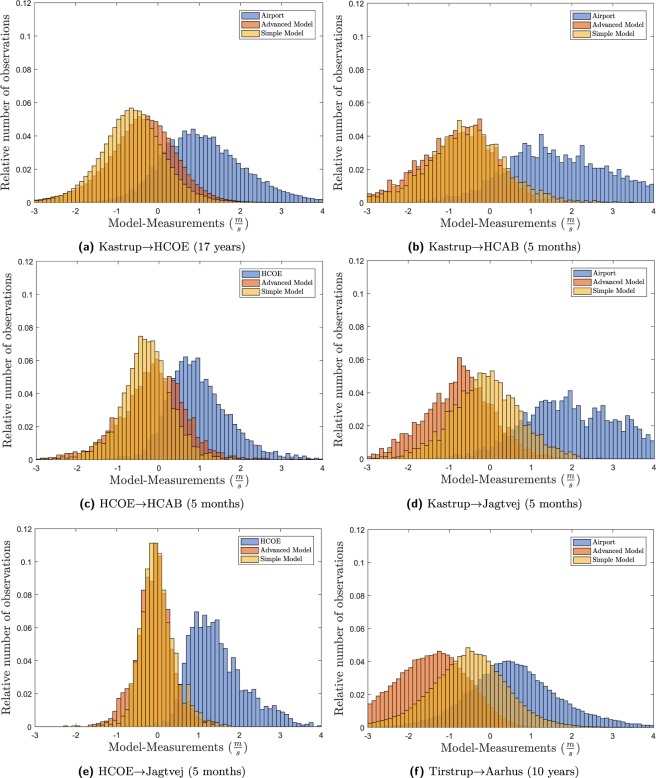
Histograms of respectively the input minus the measurements or the models minus the measurements.

The histograms of the residuals between the input and the measurements and the results from the models and the measurements can be seen in Fig. 6. In general, the models are having smaller and more narrow bias compared with the input. Figure 6 shows mixed results with the advanced model being a little bit better for Kastrup→HCOE and HCOE→HCAB, only small differences between the two models for Kastrup→HCAB and HCOE→Jagtvej, whereas the simple model performs better for Kastrup→Jagtvej and Tirstrup→Aarhus. In general, this is in line with the results from the validation against median values where the reasons have also been discussed.

As can be seen, the longer the extrapolation distance the wider the distributions. This is again caused by turbulence in the atmosphere on all length scales between the two stations. Reducing the spread further will be challenging given that this turbulence is not measured in the present project.

## Conclusion

The upwind terrain influences the modelling of the urban wind speed to a large extent. Modelling this effect is a significant challenge which in the present paper has been met to some extent. The model results show (except Aarhus) good agreement between the models and the measurements compared to model results given in e.g.^16^ for an atmospheric dispersion model.

The influence of the upwind terrain was shown to be overestimated in the model, and future work should aim at reducing this effect. Considering that the model contains many uncertain parameters, this result is acceptable.

The agreement in wind direction between the stations were shown to be high. Moreover, the influence of atmospheric stability, horizontal temperature gradients, land-sea breeze, temperature, global radiation, and Monin-Obukhov Length were shown to be small. One remaining question is whether this finding is only valid for the present location or it represents a more general phenomenon.

Many alternative parametrizations to the ones selected in the present study exist. To direct future research to the parametrizations with the largest influence on the model, sensitivity analyses, in line with the ones presented in^16^, could be performed. This could subsequently form the basis for studies on the comparison of the available parametrizations. Likewise, comparison studies between different types of models (e.g. numerical weather presiction models, multivariate linear regression models etc.) would be relevant to highligt strengths and weaknesses of different modelling approaches.

In the present study, the urban areas are assumed to be flat although the height above sea level varies by several tens of meters. The influence of the urban topography is likewise a potential area for a future study.

Modelling the wind speed in the urban canopy layer is complicated and several parametrizations has been proposed in the literature. One remaining question is the validity of these parametrizations, where measurements in the canopy layer, wind tunnel studies, and the use of CFD (Computational Fluid Dynamics) models could contribute to answer this question.

## Methods

In the analyses of the present study, wind speeds below 2 m s^−1^ have been removed (with the exception of the classification into atmospheric stability classes). This was done to avoid the difficulties of the anemometer start up speeds, and since these data will be disproportionately influenced by turbulence. Moreover, the given uncertainty of 0.1 m s^−1^ on the cup anemometers amounts to 5% at 2 m s^−1^ (see Supplementary Material for more information). This means that the measurement uncertainties will be high for these data and they are thus excluded. Data above 10 m s^−1^ are likewise excluded to reduce calculation time. This is not expected to influence the results, since these data constitute a very small fraction of the total data.

### Measurements

Long term wind speed measurements have been performed in Copenhagen, Denmark at the H. C. Oersted Institute, Copenhagen University (HCOE) and in Aarhus at the town hall under the Danish Air Quality Monitoring Programme for the period 1994 to 2010 for HCOE and for the period 2001 to 2010 for Aarhus^38^. The details of the meteorological instruments used in the present study can be found in Table 1 in the Supplementary Material. The cup anemometers are located on 7 m masts on urban rooftops. This is referred to as the *mast* type measurements in Table 1 in the Supplementary Material. The uncertainty in the wind speed measurements is described in the Supplementary Material. The wind direction is measured using two types of wind vanes (Risoe P2633A or Vector Instruments W200P) with a wind direction resolution of 0.2° and a specified uncertainty of ±3° (in steady winds over 5 m s^−1^). The data scanning frequency of both the wind speed and the wind direction measurements is 0.1 Hz. These data is subsequently averaged to hourly wind speeds and directions. The data is subsequently quality controlled using the procedure described in^16^.

For the present study, a wind measurement campaign was designed. The campaign consisted of setting up two sonic anemometers on rooftops in Copenhagen, Denmark at respectively Jagtvej and H. C. Andersens Boulevard (HCAB) to measure the wind speed close to the roof level. The sonic anemometers were mounted 4 m to 5 m above roof level. This is referred to as the *roof* type measurements in Table 1. This height was chosen in line with the aim of the study being to measure the wind speed in the roughness sublayer. The measurements were performed over a five months period from December 20, 2014 to May 1, 2015. The two locations were chosen, since extensive air pollution studies at the two streets have been performed in the past (e.g.^44–46^). The sonic anemometer at Jagtvej was a RM Young 81000 with a specified accuracy of ±1% or ±0.05 m s^−1^ below 30 m s^−1^ and ±3% above 30 m s^−1^, and the sonic anemometer at HCAB was a Metek USA-1 with a specified accuracy of 0.1 m s^−1^ or 2% at 5 m s^−1^. Both sonics were sampling at 10 Hz. The measurements were despiked by removing measurements with accelerations larger than 5*σ* (where *σ* is the standard deviation of the hourly average) or velocities larger than 10*σ*. Following the recommendation from^47^, no rotations were applied to the sonics. This is because, the flow in the urban roughness sublayer is highly three-dimensional. The assumptions for the rotation techniques are therefore not fulfilled. The measurements were subsequently averaged to hourly values with the wind speed being the sum of the *u*, *v*, and *w* components.

The wind speed measurements from Kastrup Airport and Tirstrup Airport were obtained from National Climatic Data Center, National Oceanic and Atmospheric Administration (ncdc.noaa.gov). This means that the measurements were not performed by the authors but represent standard meteorological data publicly available. The wind speeds were obtained as METeorological Aerodrome Report (METAR) measurements. These have a wind direction resolution of 10° and a wind speed resolution of 0.45 m s^−1^ (1 mile/h). These were subsequently averaged to hourly wind speeds and directions. The details of the measurements are summed up in Table 1, and the location of the measurement stations can be seen in the attached Google Maps file.

### Wind speed model

The wind speed model should simulate the hourly mean wind speed at the height and location of interest, in line with the approaches used for the regulatory air pollution models. This is a different approach compared with all the studies in Table 4 in the Supplementary Material, in that these are modelling various forms of area-averaged winds. Because of the three-dimensional nature of the urban roughness sublayer, both the measurements and the modelled wind speed cannot be claimed to be representative of a wider area. The input to the model should come from a measured wind speed either at a non-urban location, e.g. an airport, or an urban mast. As a general principle, all parametrizations are applied in the model with the published parameters in order not to add increased parameter uncertainty to the model.

#### Extrapolation scheme

The wind speed is extrapolated from one location to another using the principle by^31^ as follows:

1. The “macrowind” (*S*_*h*_), also called the “effective geostrophic wind”, is calculated from a measured wind speed through an upwards vertical extrapolation. It should be emphasised that the macrowind is not necessarily equal to the synoptic geostrophic wind speed derived from macroscale pressure gradients^31^.
2. The speed and direction of the macrowind are assumed to be constant over the area of extrapolation thus allowing horizontal extrapolation.
3. The urban wind speed is calculated from the macrowind through a downwards vertical extrapolation.

#### Model summary

The model can be summarised in the following steps:

1. The *Geographical preprocessor* generates a map of *z*_0_ and *d* as a function of wind direction. The input is maps of land use and building geometry in raster format. This procedure is described in the Supplementary Material.
2. The upwind distances from the station to the onset of the IBLs are calculated from the map of *z*_0_, as described in the Supplementary Material.
3. The heights of the individual IBLs (*δ*), at the location of the station, are calculated using the approach of^48^.
4. The blending height is calculated based on the distance to the onset of the closest IBL to the station.
5. The upwind footprint of the mixed layer is calculated as described in the Supplementary Material.
6. The boundary layer height (*h*_*ubl*_) is calculated using the approach of^49^.
7. *S*_*h*_ is calculated with repeated using the approach of^31^ (upwards extrapolation).
8. *S*_*h*_ is assumed to be constant between the two locations thus providing horizontal extrapolation.
9. The process is repeated for the downwards extrapolation.
10. The results are subsequently averaged over a 30° wind direction interval to account for hourly wind direction meandering.

Details of the calculations in the individual step can be found in the Supplementary Material. For the downwards extrapolation, Items 5 to 7 are iterated since *h*_*ubl*_ is a function of the wind speed. The above described wind speed extrapolation model was implemented in Matlab by the authors. Differences between the present modelling approach and that of previous studies are discussed in the Supplementary Material.

#### Model without influence of the upwind terrain

To analyse the influence of the upwind terrain, a simpler model was developed based on the same principles as described above. The simple model also uses the extrapolation scheme from^31^ but applies several simplifying assumptions:

- *z*_0_ and *d* are calculated from the building height of the building where the anemometer is located (*h*_*r*_) rather than using the geographical preprocessor. The equations for *z*_0_ and *d* are simplified expressions of^50^ as follows:
  1 $${z}_{0}=0.15{h}_{r}$$
  2 $$d=0.7{h}_{r}$$
  It is recommended that the maximum allowed building height for the parametrisation is 20 m^50^. This recommendation has been implemented in the model. The airport stations are assigned a constant roughness length of 0.005 m (short grass) following^51^. The vertical extrapolation is performed with one roughness length and displacement height instead of using the above described IBL approach. This means that upwind influence on the station is not taken into account.
- The horizontal extrapolation height is assumed to be 4*h*_*r*_ following the recommendation by^52^ thus also removing the wind speed dependency of the model.

This approach is referred to as the *simple model* in the results section.

## Supplementary information

Google Maps of the Stations

Supplementary Material
