# Supplementary material for: Micro-scale modelling of the urban wind speed for air pollution applications
Source: Sci Rep. 2019 Oct 3;9:14279. doi: 10.1038/s41598-019-50033-2 (PMC6776543; doi:10.1038/s41598-019-50033-2)
Supplement: Supplementary file 2 — Supplementary Material [file 41598_2019_50033_MOESM2_ESM.pdf]

Supplementary material for Micro-scale modelling of the urban wind speed for air pollution applications

Thor-Bjørn Ottosen[1,2,3], Matthias Ketzel[4], Henrik Skov[2,4], Ole Hertel[4], Jørgen Brandt[4], and Konstantinos E. Kakosimos[3*]

[1]Institute of Science and the Environment, University of Worcester, Worcester, UK
[2]Department of Chemical Engineering, Biotechnology and Environmental Technology, University of Southern Denmark, Odense, Denmark
[3]Department of Chemical Engineering, Texas A&M University at Qatar, Doha, Qatar
[4]Department of Environmental Science, Aarhus University, Roskilde, Denmark
[*]corresponding.k.kakosimos@qatar.tamu.edu

Measurements

The details of the meteorological instruments used in the present study are summarized in Table 1. The specified uncertainty on the cup anemometer wind speed measurements is about $0.1\,\frac{\mathrm{m}}{\mathrm{s}}$ below a wind speed of $10\,\frac{\mathrm{m}}{\mathrm{s}}$ and $1\,\%$ of the wind speed readings from $10\,\frac{\mathrm{m}}{\mathrm{s}}$–$55\,\frac{\mathrm{m}}{\mathrm{s}}$. The RISOE anemometer (used at the two mast locations) was examined for overspeeding and that the overspeeding could reach a level between $1.5\,\%$–$2.0\,\%$ was found[1]. A cup anemometer with a distance constant of $1.5\,\mathrm{m}$ with a sonic anemometer at various levels in an urban environment was compared[2]. With a few exceptions, the effect of overspeeding was on average found to be smaller than $0.1\,\frac{\mathrm{m}}{\mathrm{s}}$. This number is thus used as the cup anemometer uncertainty in the present study.

Measurements at similar heights to the present study has been done[3–6]. Of these, only[3] have considered the influence of the roof and the nearby buildings through analysing the wind direction dependency of the streamline deflection and the turbulence intensity plus a wind tunnel study[7]. The influence of the roof and nearby buildings on a roof-level measurement as a function of height is thus an ongoing research topic. In the absence of such data (e.g. a wind tunnel study for the specific area, multiple anemometers etc.), the influence of the roof and nearby buildings were examined in the present study by plotting boxplots of the streamline deflection and turbulence intensity as a function of wind direction using the same procedure as[3]. This analysis showed only small effects of the roof and nearby obstacles. The mean and standard deviation of the streamline deflection (ψ) and the turbulence intensity (T) for the two sites can be found in Table 1.

Discussion of model assumptions

Depending on the distance and the topography between the stations, the assumption of unchanged wind direction at macrowind height may not always be valid. However, developing a model for the changing wind direction of the macrowind was considered beyond the scope of the present study.

Following the arguments of[8], the macrowind can be assumed to be constant when the following criteria are fulfilled:

- Stationary climate – the study area is characterised by a steady isobar pattern.
- The convection is fairly homogeneous across the region.
- The topographic effects are small.

The measurement stations used in the present study are located in Denmark between 54°–58° N and 8°–13° E. As seen from Table 1 and the attached Google maps file, the distance between the stations is thus much smaller than the size of typical atmospheric pressure systems. The isobar pattern can thus be assumed to be homogeneous across the region. This was also found by[9] from a barometer campaign across the region. Similarly, the day-to-day variations

Station:	HCOE	Jagtvej	HCAB	Kastrup	Aarhus	Tirstrup
Type:	Mast	Roof	Roof	Airport	Mast	Airport
Wind speed instrument:	Risoe P2546A cup anemometer or Vector instruments A100 cup anemometer	RM Young 81000 sonic anemometer	Metek USA-1 sonic anemometer		Risoe P2546A cup anemometer or Vector instruments A100 cup anemometer	
Wind speed resolution ($\frac{m}{s}$):	0.05	0.01	0.01	0.45	0.05	0.45
Startup speed ($\frac{m}{s}$):	0.2–0.4	0.0	0.0		0.2–0.4	
Distance constant (m):	1.8–2.3				1.8–2.3	
Height above ground (m):	32.7	22.9	25.3	10	16.5	10
Roof height (m)[a]:	25.7	18.6	20.0	0	9.5	0
Years:	1994–2010	2014–2015	2014–2015		2001–2010	
Measurements (h):	122 005	2040	3270		57 962	
Streamline deflection ψ (°):		-0.5±2.7	7.0±3.4			
Turbulence intensity T:		0.46±0.09	0.43±0.08			
TI Range:		[0.23:0.91]	[0.15:1.79]			
Distance to other stations (km):						
Kastrup:	11.0	11.1	8.2			
Jagtvej	0.5					
HCAB:	3.0					
Aarhus:						32

Table 1. Overview of the measurements used in the present study. The number of hourly measurements is after quality control. The streamline deflection (ψ) and turbulence intensity (T) are given as the mean ± the standard deviation of the hourly dataset.

[a] Calculated using the building height calculation procedures described in ?? in the supplementary material for consistency.

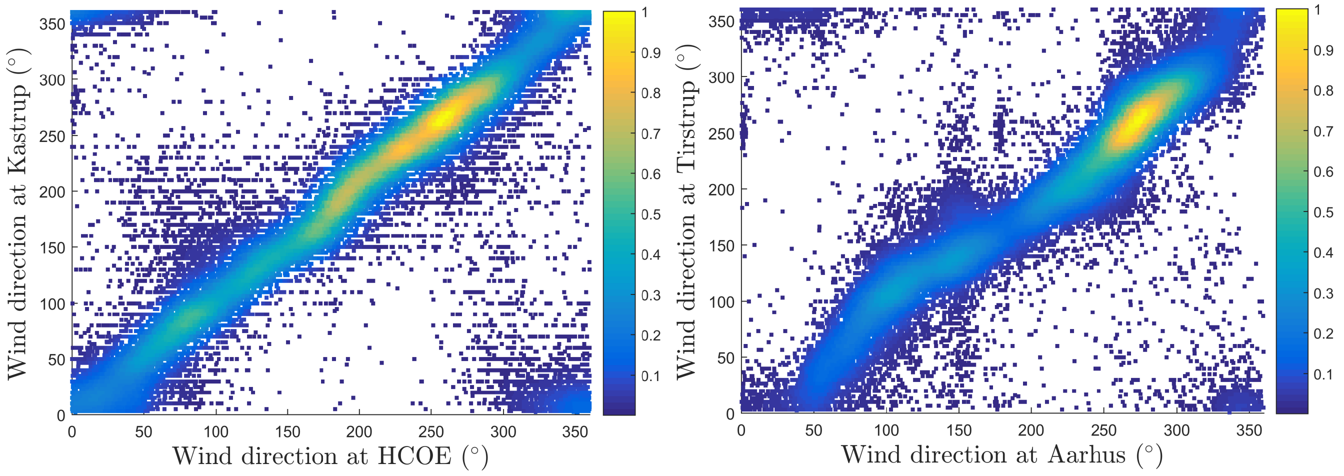

(a) Copenhagen **(b)** Aarhus

Figure 1. Scatterplot of the wind directions between Kastrup and HCOE. The colors represent relative density of points.

can be assumed to be small and the diurnal cycle homogeneous across the region.

The spatial inhomogeneity in the convection across the region has, to the best of the authors knowledge, not been examined previously. However, it is assumed to be homogeneous because of the short distance between the stations. Moreover, the winds are often strong in Denmark thus convection is expected to play a minor role.

The area under study has negligible topographic effects. It is therefore plausible that the region of study is particularly well suited for this kind of wind speed extrapolation. To the best of the authors' knowledge, studies of the influence of urban topography on the urban wind speed on this scale ($< 10\,\text{km}$) have not been carried out.

Comparison of wind directions

To analyse the validity of the assumption of constant wind direction among the stations, a scatter plot of airport wind direction versus urban mast wind direction for HCOE is shown in Fig. 1a and for Aarhus in Fig. 1b. The two airport-mast station pairs have the longest extrapolation distances cf. Table 1. This should make any wind direction deviation more obvious. For HCOE there is good agreement and no systematic bias between the measurements from the two sites. This is also confirmed by the circular correlation coefficient being 0.94. For Aarhus the agreement is less pronounced especially in the interval $[0° : 50°]$. The circular correlation coefficient is 0.86 for Aarhus. This indicates that the assumption of constant wind direction is degrading with extrapolation distance.

Influence of atmospheric stability

To analyse the influence of atmospheric stability on the extrapolation scheme, the relative wind speed for the two airport-mast pairs were classified according to the scheme by[10]. The classification was based on the measured wind speed and cloud cover in the airport and the global radiation measured at the urban station since all three quantities were not available at the same location. This off course adds an element of uncertainty to the classification.

As can be seen, the measurements are heavily dominated by neutral and slightly unstable atmospheric stability, and the distribution for Aarhus is skewed. The skewness for Aarhus is first and foremost because of the low airport wind speeds giving rise to disproportionately high relative wind speeds. Since the model is aiming to reproduce the most common wind speed, the median is used in the following comparison. For Copenhagen, the median value of the relative wind speed for all the measurements is 0.80, whereas the median value for Class D is 0.77 and for Class C 0.84. For Aarhus the median value of the relative wind speed for all the measurements is 0.86, whereas the median value for Class D is 0.82 and for Class C 0.90. This indicates that the influence of atmospheric stability is comparable to the measurement uncertainty and thus can be regarded as a small effect for the present study.

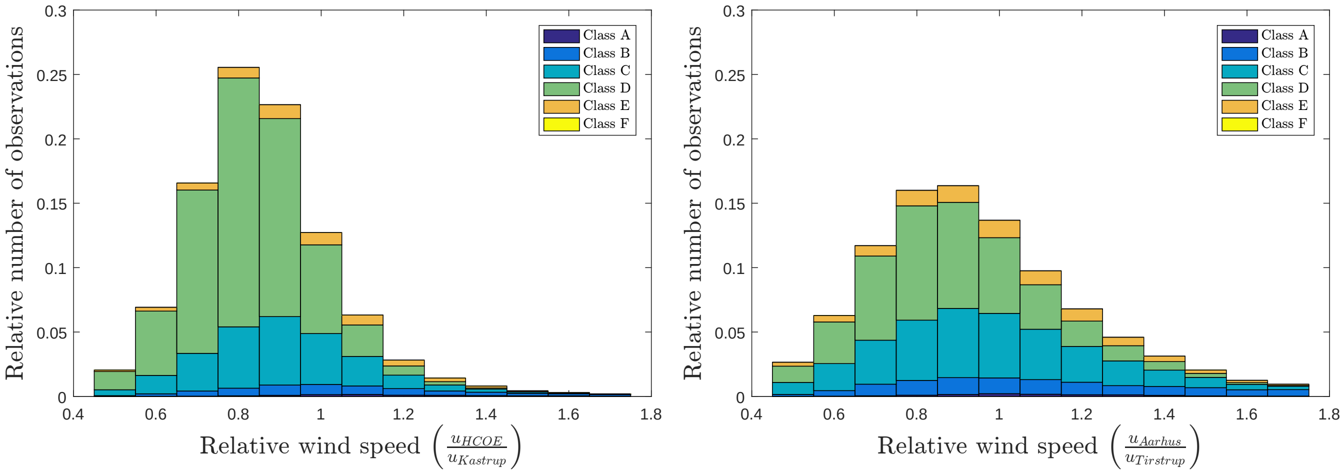

(a) Copenhagen

(b) Aarhus

Figure 2. Stacked bar charts of the relative occurrence of the atmospheric stability classes. Class A = extremely unstable; Class B = moderately unstable; Class C = slightly unstable; Class D = neutral, Class E = slightly stable; Class F = moderately stable.

Applicability of the log-profile

In the present model, the log-profile is applied to the entire atmospheric boundary layer despite only being valid in the inertial sublayer. According to[11], the log-profile is valid up to roughly 200 m whereas[12] stated it to be valid for 100 m or more. However, there is at present not compelling evidence to suggest that the alternative expressions for the profile, being the power law profile or the Deaves and Harris profile[13], should perform better. Moreover, the logarithmic profile has been used for this type of extrapolation in several previous studies for e.g. wind energy and air quality[14–20]. Thus for simplicity, Eq. (8) is used for the vertical extrapolation in the present study.

Details of the modelling procedure

Geographical Preprocessor

The spatially distributed roughness length and displacement height was modelled based on input data from a geographical information system (GIS). Based on a visual inspection of the GIS data, it was seen that certain land use classes dominated the areas under inspection. To limit the number of land use classes in the model, a number of general land use classes were used:

- *Parks* covers the base layers of recreational areas, urban forests, and cemeteries.
- *Forests* only covers rural forests.
- *Airports* cover the areas not occupied by other land use classes in the airport maps.
- *Unclassified urban* cover the areas not occupied by other land use classes in the urban maps such as parking lots, railway areas etc.

The roughness length from the buildings depends on the building height and geometry, as described below. The fixed roughness lengths for these land use classes are summed up in Table 2. The value of 0.35 m for the roughness length of parks was calculated based on the morphology of the buildings in the same 1 km × 1 km as the park. This means that the value does not represent an explicit parametrization of the park roughness (Padhra, personal communication). Modelling urban parks as having a fixed roughness length is questionable. The roughness length will obviously be different for an urban forest and a park consisting of short grass. However, in most cases the detailed information about the kind of park is not available. Moreover, there is, to the best of the authors' knowledge, no methodology devised in the literature on how to model low roughness parks versus high roughness parks.

The information on building heights was obtained from laser scanning data resulting in two Digital Elevation Models (DEMs) respectively with and without buildings. The DEMs cover all of Denmark on a 1.6 m × 1.6 m spatial

Land use class:	z_0(m)	Source:
Water	$0.01 \cdot 10^{-2}$	[21]
Airport (short grass)	$0.50 \cdot 10^{-2}$	[21]
Parks	0.35	[22]
Forests	1.00	[21]
Unclassified urban	0.10	

Table 2. Table of roughness lengths for the land use classes modelled by a fixed roughness. The displacement height for all the land use classes is 0 m.

resolution which was subsequently resampled to 5 m × 5 m spatial resolution to limit the amount of calculations. The data were obtained from the Danish Geodata Agency (http://eng.gst.dk/). The information on the spatial extent of the urban water bodies, forests, and cemeteries were obtained as vector data likewise from the Danish Geodata Agency (http://eng.gst.dk/). For Copenhagen the information on the recreational areas was obtained in a similar manner. These data were not available for Aarhus, and the recreational areas were therefore digitised by hand based on a visual inspection of Google Earth.

The roughness length and displacement height of the areas covered by buildings depend on the building height and geometry. A model for the relationship among the frontal area index (frontal area of buildings divided by ground area) (λ_f) on the one hand and z_0 and d on the other hand was set up[23]. This model builds on the data of several earlier studies and was therefore used in the present study:

$$z_0 = H_r \lambda_f \quad \text{for} \quad \lambda_f < 0.15 \tag{1}$$
$$z_0 = 0.15 H_r \quad \text{for} \quad \lambda_f > 0.15 \tag{2}$$
$$d = 3\lambda_f H_r \quad \text{for} \quad \lambda_f < 0.05 \tag{3}$$
$$d = H_r(0.15 + 5.5(\lambda_f - 0.05)) \quad \text{for} \quad 0.05 < \lambda_f < 0.15 \tag{4}$$
$$d = H_r(0.7 + 0.35(\lambda_f - 0.15)) \quad \text{for} \quad 0.15 < \lambda_f < 1.0 \tag{5}$$

Where H_r is the building height. According to[23], the above equations should not be used for areas with an average building height larger than approximately 20 m. Therefore, an artificial maximum average building height was incorporated in the model. To calculate the building height, the DEM without buildings was averaged on a 100 m × 100 m resolution. This resolution is in the same order of magnitude as the size of the buildings in the city. The DEM without buildings was subsequently subtracted from the DEM with buildings.

All tessellations were designed to have the location of the receptor in a cell center. The averaging of the DEM without buildings corresponds to levelling the city in the model.

The resulting DEM of building heights was subsequently divided into cells of 50 m × 50 m, a resolution decided based on a visual inspection of the map. The average building height of each of these supercells was subsequently calculated based on an area-weighted average.

Although the urban roughness length calculated on 50 m × 50 m resolution has high spatial variability, the z_0 and d experienced by the wind changes more slowly. To reflect this in the model, the cities under consideration were divided into neighbourhoods based on the roughness. λ_f and H_r was subsequently calculated for each neighbourhood instead of each cell. Following the approach of[24] the unit vector perpendicular to each surface of the DEM was calculated in Matlab. All vectors were subsequently subjected to the dot product with a horizontal unit vector in the direction of the wind direction. This gives the projection of the building surface along that direction. Lastly these results were summed over all negative pixels to get the frontal area index as a function of wind direction. This procedure was done for each 5° of wind direction as a balance between precision and calculation time. The neighbourhood delimitation was done through a thresholding procedure. The thresholds were set iteratively to obtain reasonably sized neighbourhoods with relatively homogeneous statistical properties. The following threshold

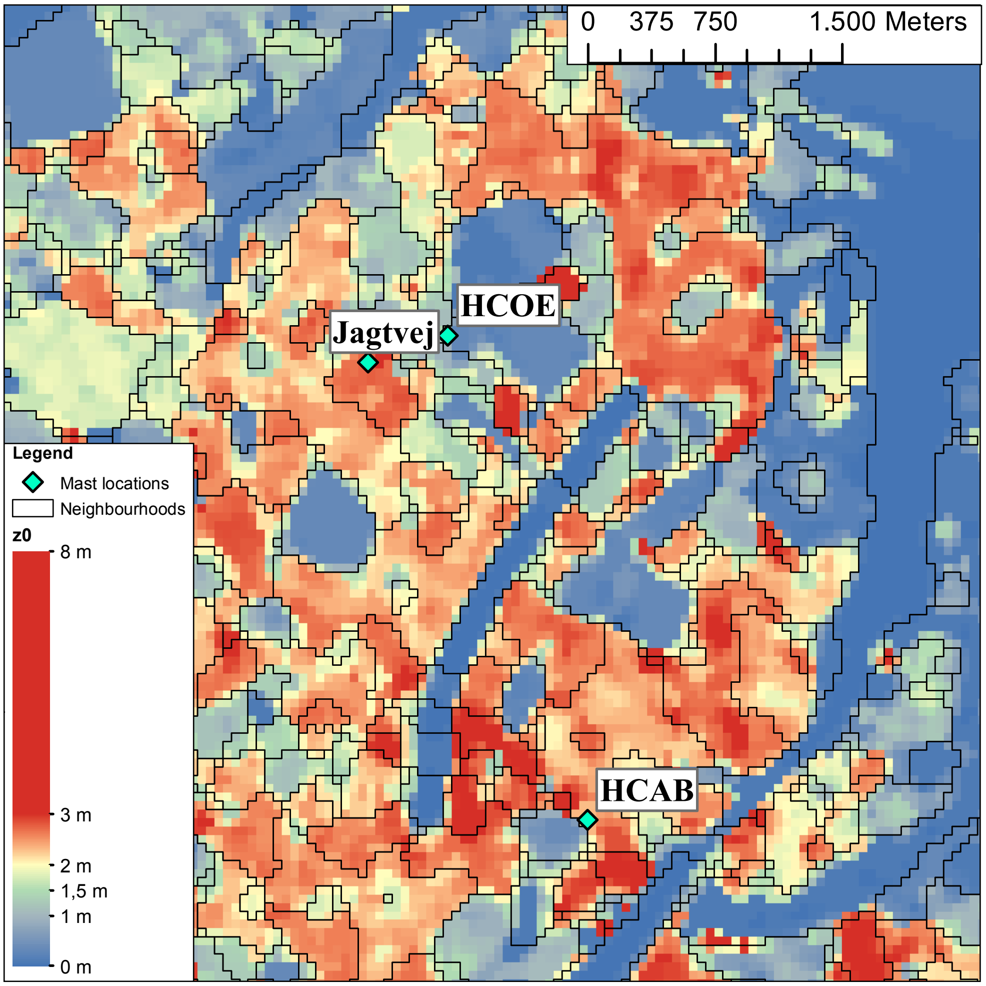

Figure 3. Map of the neighbourhood delimitation procedure. The figure illustrates that the neighbourhoods (found by the algorithm) matches fairly well with areas of homogeneous roughness

criteria was used:

$$\frac{\sigma}{\mu} < 0.25 \qquad \text{for} \qquad z_0 < 1.5\,\text{m or } z_0 > 3.0\,\text{m} \tag{6}$$

$$\frac{\sigma}{\mu} < 0.05 \qquad \text{for} \qquad 1.5\,\text{m} < z_0 < 3.0\,\text{m} \tag{7}$$

Where σ is the standard deviation and μ is the mean value of the roughness length of the part of the map within the threshold. The constants were chosen to give reasonably sized neighbourhoods. The limits were set tighter in the interval 1.5 m–3.0 m to obtain smaller neighbourhoods in the urban areas, thereby obtaining a higher spatial resolution close to the receptor. An example of the results of the neighbourhood delimitation algorithm for a wind direction of 0 ° is shown in Fig. 3.

The above procedure for calculating z_0 and d from GIS data was implemented in Matlab by the authors.

Algorithm to detect IBLs from roughness maps

The model utilizes an automatic procedure to determine IBLs from roughness maps. This algorithm proceeds through several steps:

1. A table of values for roughness length and displacement height for each meter against the wind direction from the station to an upper integration limit (being 5 km) is generated numerically from the maps. Neighbourhood

borders will then be represented as a step change in this table.

2. The step changes, and the distances to each step change, in roughness length and displacement height is generated from this table.

Subsequently the step changes are averaged based on several assumptions[23]:

- Only step changes larger than a factor of two generate IBLs. Smaller step changes are averaged weighted with their length upwind along the wind direction.
- Only step changes with an upwind length greater than 500 m or 10 times the average height of the roughness elements generate IBLs.
- When an IBL has reached a length of more than 1000 m, a new IBL is started at the next step change in roughness regardless of the size of the step change.

The above assumptions are made to make sure the surface length of the individual IBL has a reasonable size. The values of 500 m and 1000 m are chosen based on the developers experience with the model to generate reasonably sized IBLs. It is moreover assumed that IBLs with height above the blending height or below the receptor height do not influence the wind speed at the receptor.

Vertical extrapolation

The wind speed can be vertically extrapolated at a specific location from the measured wind speed (U), measured at height h, through a logarithmic wind speed dependency [25, p. 768]:

$$U_{\text{top}} = U \frac{\ln\left(\frac{h_{\text{top}} - d}{z_0}\right)}{\ln\left(\frac{h-d}{z_0}\right)} \tag{8}$$

Where U_{top} is the extrapolated wind speed, d is the displacement height, z_0 is the roughness length, and h_{top} is the height of the vertical extrapolation. This approach is also applied by all the references in Table 4. In the present study z_0 and d are calculated based on a GIS-procedure as described above.

The log-profile can be expanded to include the effect of atmospheric stability. The influence of atmospheric stability on the measurements is analysed above, and it is shown to be a small effect. This assumption is also fortified by[23] stating that the atmosphere in urban areas can be assumed to be neutral. Moreover, for high wind speeds (neutral stability) stability corrections will be unimportant, whereas for low wind speeds the importance of atmospheric stability in absolute magnitude will be small[26]. Lastly, following the arguments of[27], any departure from logarithmicity will be found in both the upward transformation (step 1) and in the downwards transformation (step 3) and will to a large extent cancel out. Lastly, as seen from Table 1, the measurements to directly calculate the Monin-Obukhov length are not available in the present project which hampers the development of a model that is dependent on atmospheric stability. Due to these reasons, the simple log-profile in Eq. (8) was used in the present model. Future work should aim at directly measuring the atmospheric stability and variables to predict it in urban areas thus to allow a future model to include this effect. A discussion of the applicability of the log-profile at different heights is presented above.

Modelling the roughness sublayer

The extrapolation model by[28] consists of dividing the planetary boundary layer into two layers in the vertical direction. In[28], Eq. (8) is applied in both layers with different values for z_0 and d in the two layers. The two profiles are then patched together at the *blending height* (h_{bl}) being the limit between the two layers. The present study also divides the boundary layer into a mixed layer and a roughness sublayer. The two profiles are subsequently patched together at the blending height. As opposed to[28] the present study models z_0 and d as being dependent on the upwind terrain from the station both at the input station and at the receptor station, as illustrated in Fig. 5. The details of the calculation of the blending height can be found below.

In the present model, the presence of the inertial sublayer is ignored for simplicity. This is a reasonable assumption since the inertial sublayer is a thin layer for rough surfaces as e.g. urban areas[29]. In[30], a procedure for the calculation of the wind speed in the roughness sublayer is described. However, this procedure requires that the Reynolds stress is determined within the roughness sublayer or that this can be estimated from the measured variables. The Reynolds

stress is not directly measured in the long term campaigns of the present project, and the procedures for estimating it from measured variables are highly uncertain. Instead, the roughness sublayer is subdivided into a number of internal boundary layers (IBL) with different roughness length and displacement height. Internal boundary layers form when the wind is passing borders between neighbourhoods with markedly different roughness and displacement height. Within each internal boundary layer, the wind speed is assumed to be in equilibrium with the corresponding ground surface, an approach also adopted by[31,32]. This means that Eq. (8) can be applied in each IBL and be patched together at the interface. Above the IBL, the upstream roughness length and displacement height will be dominant, whereas below the IBL the downstream roughness length and displacement height will be dominant. The vertical wind speed profile in the presence of IBLs is illustrated in Fig. 5. This approach has been widely applied when modelling the flow from rural to urban areas but has not had widespread application on smaller scale.

The heights of the IBLs are parametrised in the model using the following formula ([33] also used in[34]):

$$\delta_i = 0.28 z_{0,i} \left(\frac{x_i}{z_{0,i}} \right)^{0.8} \qquad i = 1, 2, \ldots \delta_i = h_{bl} \tag{9}$$

Where δ_i is the IBL height of IBL number i, and x_i is the distance from the station to the start of IBL number i. δ_i, $z_{0,i}$, and x_i are illustrated in Fig. 4.

The upwind influence on the station is then modelled by detecting the upwind IBLs from roughness maps along a straight line starting at the station and moving against the wind direction. The upwind influence is stopped when the height of the most upwind IBL equals the blending height, as illustrated in Fig. 4. The procedure for detecting IBLs from the roughness maps is described above. A number of expressions for the IBL height and the available wind-tunnel measurements of this was reviewed in[35]. In the future, comparisons of the different parametrizations should be performed.

Calculation of the blending height

Parametrizations for calculating the blending height are available in the literature[36,37] but since none of these approaches are unambiguous in their calculation, a simpler empirical approach was adopted.

The blending height is set to $0.05x_1$[1]; x_1 being the length measured along the surface of the IBL closest to the receptor, as illustrated in Fig. 4. The value of 0.05 is chosen to give reasonable model performance, but it is in the range of values presented by[38].

Modelling the mixed layer and the canopy layer

The mixed layer is also modelled using Eq. (8) with a wind direction dependent roughness length and displacement height. The roughness length and displacement height for the mixed layer (see Fig. 4) was calculated as a function of the upwind roughness length and displacement height following[39]:

$$\left[\ln \left(\frac{h_{bl}}{z_0^{\text{eff}}} \right) \right]^{-2} = \frac{1}{x_{\text{end}} - x_{\text{start}}} \int_{x_{\text{start}}}^{x_{\text{end}}} \left[\ln \left(\frac{h_{bl}}{z_0(x)} \right) \right]^{-2} dx \tag{10}$$

Where h_{bl} is the blending height, z_0^{eff} is the roughness length of the mixed layer, $z_0(x)$ is the spatially varying roughness length, and x_{start} and x_{end} are the upwind integration limits. x_{start} and x_{end} could be determined from a footprint model. However, setting up an entire footprint model was deemed outside the scope of the present project. Moreover, whereas footprint models for vegetation canopies exist no such models have been developed for urban areas[30]. The model of[40] was tested but performance decreased. Instead a simpler empirical approach was adopted:

$$x_{\text{start}} = z_0 \sqrt[0.8]{\frac{h_{bl}}{0.28 z_0}} \tag{11}$$

$$x_{\text{end}} = z_0 \sqrt[0.8]{\frac{h_{bl}}{0.28 z_0}} + 1000\,\text{m} \tag{12}$$

Where x_{start} is obtained by setting $\delta = z_{bl}$ in Eq. (9). Following a general approach where winds higher above ground are influenced by areas farther upwind, it is natural to start the integration at x_{bl} since areas closer to the receptor

[1] In[38] x_1 is replaced by $L_p = \frac{1}{n}\sum_1^n x_i$. Since each station is only influenced by a few IBLs, the model failed to converge when implementing this expression. The present expression is therefore a simplification.

have already influenced the receptor through the IBLs. To end the integration 1000 m farther upwind is chosen to cover an area large enough to mix up several roughness lengths without getting too much upwind influence. The subsequent wind direction averaging will account for the second dimension of the footprint.

For certain wind directions, it is evident from data for Kastrup to HCOE that the wind is passing over areas of changing large-scale roughness. This is the case e.g. when passing from the urban area to the airport or from the sea to the airport. In these situations a new mesoscale boundary layer builds up at the transition. The mixed layer is thus only influenced by the area up to the start of the new mesoscale boundary layer. A similar situation can occur in the urban area. Here a transition from an area with high-rise buildings to an area of lower roughness, such as a park or a lake, will cause the build up of a new boundary layer. For these wind directions the integration distances are shortened to:

$$x_{\text{start}} = 0 \tag{13}$$

$$x_{\text{end}} = 1000\,\text{m} \quad \text{For urban conditions} \tag{14}$$

$$x_{\text{end}} = x_{bl} \quad \text{For airport conditions} \tag{15}$$

The difference between urban and airport conditions is empirically determined by inspection of the data for the relationship between Kastrup and HCOE.

For certain wind directions, some of the roof level stations are below the canopy height of the upwind buildings. To account for this, an urban canopy layer was implemented in the model. The non-urban stations are assumed to be free from building influences and are thus not influenced by a canopy layer. No wind direction dependency is assumed for the urban canopy layer, the roughness length is assumed to be 0.1 m, and the displacement height is assumed to be 0 m. Exact modelling of the flow in the urban canopy layer is complicated, and the present approach is therefore a preliminary pragmatic solution. This approach is in line with the principles for regulatory air pollution models. In the future, different parametrizations should be tested, and computational fluid dynamics models could also be used for developing more accurate parametrizations.

Modelling the horizontal extrapolation height

In the present model, the horizontal extrapolation is performed at the height of the boundary layer (h_{ubl}). This rests on the assumption that above the boundary layer, the micrometeorological variables are the same for the urban and non-urban stations, an approach also adopted by[41] and[19]. The boundary layer height is higher over urban areas compared with rural areas owing to the increased friction. Likewise, the boundary layer height is higher for an area consisting of high rise buildings compared with a residential area. Despite the difference in boundary layer height, the wind speed above the boundary layer is assumed to be the same at both locations since the wind is not changing with height. In the present model, the parametrization of this height from[42,43], with the constant from[44] derived from data from[45], is used:

$$h_{\text{ubl}} = 0.06\frac{u_*}{f} \tag{16}$$

Where u_* is the friction velocity and f is the Coriolis parameter. u_* is calculated using a logarithmic wind speed profile (Eq. (8)) for a height corresponding to the blending height and z_0 and d corresponding to the mixed layer. In this way, the model becomes wind speed dependent. It is assumed that Eq. (16) is valid at the locations of the measurements by assuming that the distance from the edge of the city to the stations is large enough that a constant value has been reached. Equation (16) is only valid for neutral stability. In[30] a number of expressions for non-neutral conditions are presented. However, all these expressions contain quantities that are not routinely measured. Equation (16) is thus used since it as a minimum captures the difference in boundary layer height among areas of high (e.g. urban) and low (e.g. airport) roughness. Future work should aim at measuring this height and variables to predict it to allow a more accurate treatment of this problem.

Model summary

An example of a model situation is illustrated in Figs. 4 and 5, and the model is summarized in Table 3. In Figs. 4 and 5; the roughness lengths, IBL heights, blending heights, and boundary layer heights are chosen for illustration. The development of the IBL heights follows Eq. (9), and the shape of the wind speed profile follows Eq. (8). As can be seen, the blending height and the boundary layer height are modelled differently in respectively the urban and the non-urban areas because of the increased mixing in the urban areas. This also means that the upwind

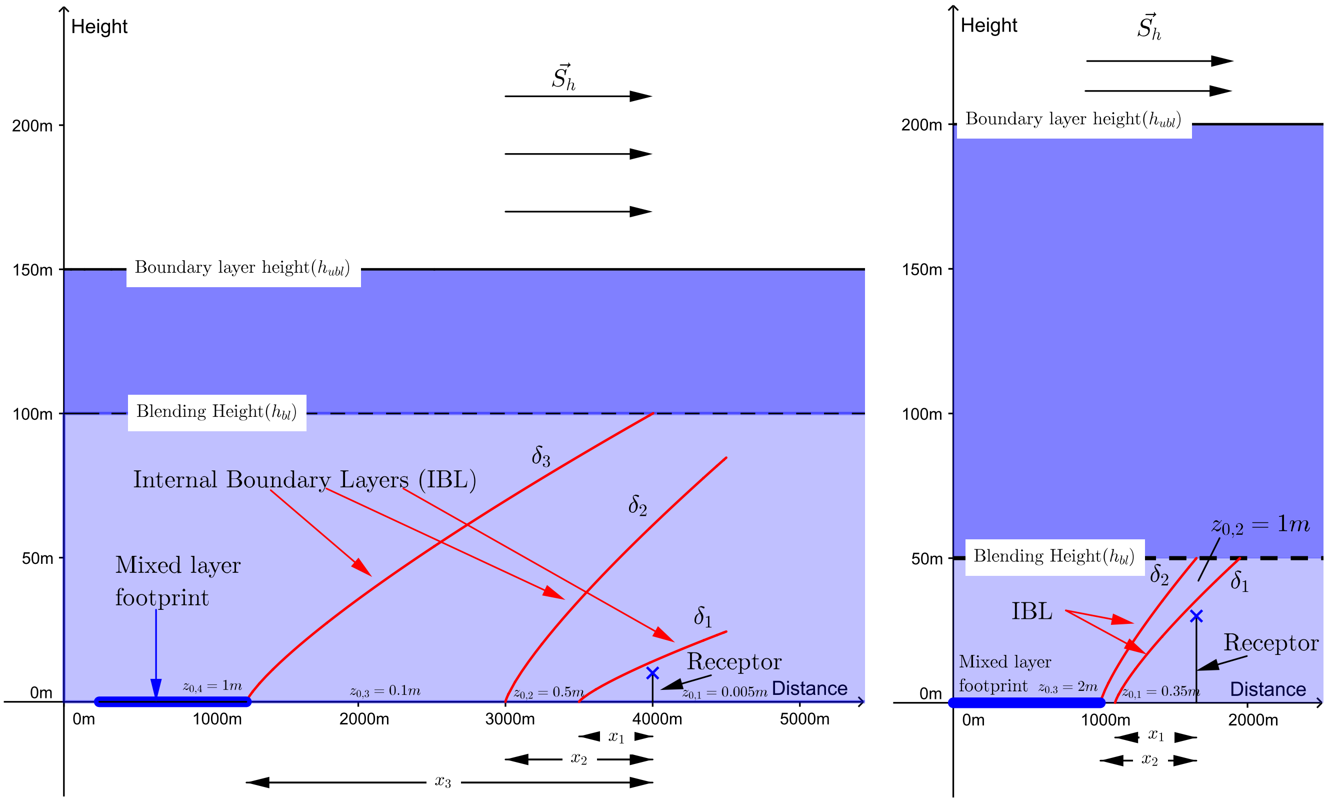

(a) Airport

(b) Urban

Figure 4. Illustration of the simplified boundary layer used in the present model for a hypothetical situation. The IBLs are marked with red, the dotted black line is the blending height, the boundary layer height is marked with a full black line, the station is marked with a blue cross, the mixed layer footprint is marked with blue, the roughness sublayer is marked with light blue, and the mixed layer is marked with dark blue.

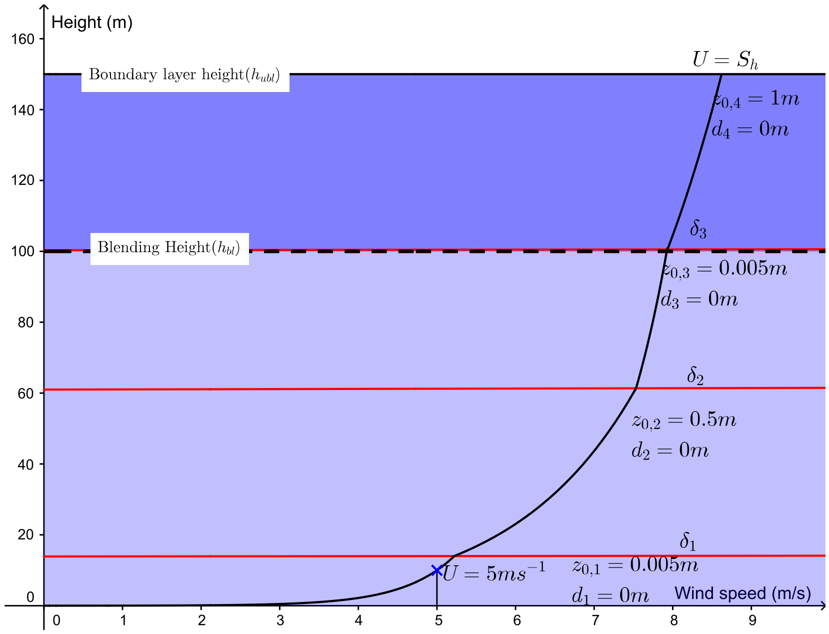

Figure 5. Illustration of the vertical velocity profile in an airport under inhomogeneous roughness for an arbitrary wind speed of 5 $\frac{\mathrm{m}}{\mathrm{s}}$. The height profile corresponds to the situation termed “Airport” in Fig. 4. The colours are used in the same way as in Fig. 4.

Description:	Source:	Equation:
Equations:		
Vertical extrapolation:	[25]	$U\frac{\ln\left(\frac{h_{\text{top}}-d}{z_0}\right)}{\ln\left(\frac{h-d}{z_0}\right)}$
Horizontal extrapolation:	[28]	$S_h(\text{input}) = S_h(\text{receptor})$
Wind direction :	[28]	$WD(\text{input}) = WD(\text{receptor})$
Boundary layer height:	[44]	$0.06\frac{u_*}{f}$
IBL height:	[33]	$0.28z_0\left(\frac{x}{z_0}\right)^{0.8}$
Blending height:	See supplementary material	$0.05x_1 \qquad h_{bl} \in [50\,\text{m} : 200\,\text{m}]$
Wind direction integration:	[46]	$\frac{1}{30}\int_{WD-15}^{WD+15} u(WD)dWD$
Integration of roughness lengths:		
Roughness sublayer:		Simple mean
Mixed layer:	[39]	$\left[\ln\left(\frac{h_{bl}}{z_0^{\text{eff}}}\right)\right]^{-2} = \frac{1}{x_{\text{end}}-x_{\text{start}}}\int_{x_{\text{start}}}^{x_{\text{end}}}\left[\ln\left(\frac{h_{bl}}{z_0(x)}\right)\right]^{-2}dx$
See section above for integration limits x_{start} and x_{end}.		
z_0 and d for buildings:		
z_0		$H_r\lambda_f \qquad \lambda_f < 0.15$
z_0		$0.15H_r \qquad \lambda_f > 0.15 \qquad h_r \in [0\,\text{m} : 20\,\text{m}]$
d	[46]	$3\lambda_f H_r \qquad \lambda_f < 0.05$
d		$H_r(0.15+5.5(\lambda_f-0.05)) \qquad 0.05 < \lambda_f < 0.15$
d		$H_r(0.7+0.35(\lambda_f-0.15)) \qquad 0.15 < \lambda_f < 1.0$
z_0 for other land-use types:		
Water	[21]	0.01×10^{-2} m
Airport (short grass)	[21]	0.50×10^{-2} m
Parks	[22]	0.35 m
Forests	[21]	1.00 m
Unclassified urban		0.10 m

Table 3. Summary of equations and constants in the model for the urban wind speed.

distance influencing the station is shorter in the urban areas. The situation termed *Airport* in Fig. 4 is where the mast is influenced by a neighbourhood with a roughness length of 0.5 m at an upwind distance of 500 m–1000 m and a neighbourhood with a roughness of 1 m roughly 3 km upwind. The first neighbourhood will generate an internal boundary layer, whereas the second neighbourhood will influence the mixed layer. In total three IBLs are assumed here. A similar situation, with low roughness (e.g. a park) close to the station and higher roughness further away, is illustrated in the situation termed *Urban* in Fig. 4, but here the station is only influenced by two IBLs before the blending height is reached. This is because of the generally lower blending height in urban areas and the higher roughness lengths causing Eq. (9) to increase faster.

Discussion

Despite applying the same overall modelling methodology, the studies in Table 4 differ in important details in both input and calculations. An overview of specific modelling aspects is shown in Table 5. A wind direction dependent z_0 and d above the blending height was used in[19] but it was assumed that z_0 and d was independent of wind direction below the blending height. The IBLs for four wind directions (North, South, East and West) were modelled in[31] to calculate annual mean wind speeds and the influence of the upwind terrain is discussed to some extent in[4]. The present approach is therefore different from all the references in Table 4 in that the exact wind direction, and thereby the upwind influence on the station, is taken into account both in the roughness sublayer and in the mixed layer. In this way, the present model becomes a microscale wind speed model as opposed to a general urban wind speed model. Moreover, the present study is, to the best of the authors' knowledge, the first to take the upwind influence of the input station (e.g. an airport) into account. This can partly be explained by the fact that many of the studies in Table 4 use wind speed databases, where the wind direction dependency has already been taken into account, as model input.

An approach from[35] was used in[41] for calculating the horizontal extrapolation height using a more complex input than was available for the present study. An equation from[47,48] for a boundary layer building up from the edge of the city in line with the IBL approach used in the present study for the roughness sublayer was used in[19]. The present approach is simpler since it avoids the challenge of uniquely determining the edge of the city. The accuracy of the different approaches is not known since a comprehensive comparison of the different parametrizations remains to be carried out[30].

References

1. Pedersen, T. F. Characterisation and Classification of RISØ P2546 Cup Anemometer. Tech. Rep., Risø National Laboratory, Roskilde (2003).
2. Christen, A. *Atmospheric Turbulence and Surface Energy Exchange in Urban Environments.* Ph.d.-thesis, Philosophisch-Naturwissenschaftlichen Fakultät der Universität Basel (2005).
3. Barlow, J. *et al.* Referencing of street-level flows measured during the DAPPLE 2004 campaign. *Atmospheric Environ.* **43**, 5536 – 5544, DOI: http://dx.doi.org/10.1016/j.atmosenv.2009.05.021 (2009).
4. Sunderland, K. M., Mills, G. & Conlon, M. F. Estimating the wind resource in an urban area: A case study of micro-wind generation potential in Dublin, Ireland. *J. Wind. Eng. Ind. Aerodyn.* **118**, 44–53 (2013).
5. Rotach, M. W. J. *Turbulence Within and Above and Urban Canopy.* Ph.D. thesis, ETH Zürich (1991).
6. Eliasson, I., Offerle, B., Grimmond, C. & Lindqvist, S. Wind fields and turbulence statistics in an urban street canyon. *Atmospheric Environ.* **40**, 1 – 16, DOI: http://dx.doi.org/10.1016/j.atmosenv.2005.03.031 (2006).
7. Carpentieri, M., Robins, A. G. & Baldi, S. Three-dimensional mapping of air flow at an urban canyon intersection. *Boundary-Layer Meteorol.* **133**, 277–296, DOI: 10.1007/s10546-009-9425-z (2009).
8. Abu Bakr, E. H. & Wieringa, J. A Boundary-Layer Model for the Determination of Hourly Surface Wind Characteristics in a Representatice Tropical African Region. *Boundary-Layer Meteorol.* **45**, 325–353 (1988).
9. Petersen, E. L., Troen, I., Frandsen, S. & Hedegaard, K. Danish Windatlas – A Rational Method of Wind Energy Siting. Tech. Rep. RISØ-R-428, Meteorology Section, Physics Department, Risø National Laboratory and Weather Service, Danish Meteorological Institute (1981).

Study:	City:	Time scale:	Validation:	Height (m)	Building Height (m)	Input:	Extrapolation Method:
15	Eindhoven, Netherlands,	Hourly	2 years	44.6	14.0	Airport	Profile
41	Basel, Switzerland	Hourly	1 month	22.4	14.0	Airport	Profile, TAPM[a]
31	London, UK	Annual	10 years	10.0	0.0	NOABL[b]	IBL
32	London, UK	Hourly	6 months	100–1000	21.0	Airport	IBL
4	Dublin, Ireland	Hourly	1 year	5.0–17	6–10[c]	Airport	Profile
19	Edinburgh, Leeds, Manchester, Nottingham, Warwick; UK	Climatological	0.5 years–5 years	4.0–47.6	0.0–45.0	NCIC[d] NOABL	Profile
51	38 cities, UK	Climatological	1 years–5 years	8.0–43.3	No information	NCIC	Profile

Table 4. Overview of recent studies on modelling of the urban wind speed ordered by publication date. The input to the model can be either measured wind speeds at a nearby airport or input from a wind speed database.

[a] The Air Pollution Model[49, 50]

[b] Numerical Objective Analysis of the Boundary Layer (http://www.rensmart.com/Weather/BERR). (Air flow model that accounts for topography).

[c] Numbers calculated from number of storyes

[d] National Climatic Information Center, UK Met Office (Interpolated measurements)

Model element:	[15]	[41]	[31]	[32]	[4]	[19]	[51]
Urban z_0 and d:	Measured[a]	Measured[b]	Calculated[c]	Calculated[c]	Constant[d]	CT: Calculated[e] / Constant<br>CT-MH: Calculated[e] / Calculated[f]<br>MH: Calculated[f] / Calculated[f]	Constant[g]
Nr of wind directions (z_0 and d):	0	0	4	4	0	CT: 0<br>CT-MH: 8<br>MH: 8	0
Neighbourhood size:	-	-	1km × 1km	1km × 1km	-	CT: $1km^2$ / -<br>CT-MH: 1km × 1km / 250m × 250m<br>MH: Wind sector / 250m × 250m	-
Extrapolation Height (m)	Calculated[h]	Calculated[i]	-	-	Constant[d]	CT: Constant[g]<br>CT-MH: Constant[g]<br>MH: Calculated[j]	Constant[g]
Nr of wind directions (Extrapolation height):	0	0	-	-	0	CT: 0<br>CT-MH: 0<br>MH: 8	0
IBLs	No	No	Yes	Yes	No	No	No
Blending height (m):	-	-	-	-	-	CT: Calculated<br>CT-MH: Calculated<br>MH: Calculated	No information

Table 5. Table of selected model properties from the previous studies and corresponding references. Three different modelling approaches called respectively CT, CT-MH and MH are used in[19]. Moreover, a two-step downwards extrapolation, which is why each model has two properties where relevant is used in[19].

a 52
b 53
c 54
d 55
e 39, 56
f 57
g 56
h 33
i 35
j 47

10. Turner, D. B. Workbook of Atmospheric Diffusion Estimates. Tech. Rep. 999-AP-26, U. S. Environmetal Protection Agency, Washington, DC. (1969).

11. Cook, N. J. The deaves and harris abl model applied to heterogeneous terrain. *J. Wind. Eng. Ind. Aerodyn.* **66**, 197 – 214, DOI: http://dx.doi.org/10.1016/S0167-6105(97)00034-2 (1997).

12. Holtslag, A. A. M. Estimates of diabatic wind speed profiles from near-surface weather observations. *Boundary-Layer Meteorol.* **29**, 225–250, DOI: 10.1007/BF00119790 (1984).

13. Deaves, D. & Harris, R. A mathematical model of the structure of strong winds. Tech. Rep. Report 76, Construction Industry Research and Information Association (1978).

14. Petersen, E. L., Mortensen, N. G., Landberg, L., Højstrup, J. & Frank, H. P. Wind power meteorology. part i: climate and turbulence. *Wind. Energy* **1**, 25–45, DOI: 10.1002/(SICI)1099-1824(199804)1:1+<25::AID-WE4>3.0.CO;2-D (1998).

15. de Wit, M., Stathopoulos, T. & Wisse, J. Airport wind speeds used for the design in urban environments: the eindhoven case. *J. Wind. Eng. Ind. Aerodyn.* **90**, 1289 – 1298, DOI: http://dx.doi.org/10.1016/S0167-6105(02)00258-1 (2002). 3rd European-African Conference on Wind Engineering (Part two).

16. Motta, M., Barthelmie, R. J. & Vølund, P. The influence of non-logarithmic wind speed profiles on potential power output at danish offshore sites. *Wind. Energy* **8**, 219–236, DOI: 10.1002/we.146 (2005).

17. van den Berg, G. P. Wind turbine power and sound in relation to atmospheric stability. *Wind. Energy* **11**, 151–169, DOI: 10.1002/we.240 (2008).

18. Soulhac, L., Salizzoni, P., Cierco, F.-X. & Perkins, R. The model SIRANE for atmospheric urban pollutant dispersion: part I, presentation of the model. *Atmospheric Environ.* **45**, 7379–7395 (2011).

19. Millward-Hopkins, J., Tomlin, A., Ma, L., Ingham, D. & Pourkashanian, M. Mapping the wind resource over UK cities. *Renew. Energy* **55**, 202 – 211, DOI: http://dx.doi.org/10.1016/j.renene.2012.12.039 (2013).

20. Emeis, S. Current issues in wind energy meteorology. *Meteorol. Appl.* **21**, 803–819, DOI: 10.1002/met.1472 (2014).

21. Oke, T. R. *Boundary Layer Climates* (Routledge, 1987), 2nd edn.

22. Wood, C. *et al.* Turbulent flow at 190 m height above london during 2006–2008: A climatology and the applicability of similarity theory. *Boundary-Layer Meteorol.* **137**, 77–96, DOI: 10.1007/s10546-010-9516-x (2010).

23. Hanna, S. R. & Britter, R. E. *Wind Flow and Vapor Cloud Dispersion at Industrial and Urban Sites* (American Institute of Chemical Engineers, 2002).

24. Ratti, C., Di Sabatino, S. & Britter, R. Urban texture analysis with image processing techniques: Winds and dispersion. *Theor. Appl. Climatol.* **84**, 77–90 (2006).

25. Seinfeld, J. H. & Pandis, S. N. *Atmospheric Chemistry and Physics* (John Wiley & Sons, Inc., 2006).

26. de Rooy, W. C. & Kok, K. A combined physical–statistical approach for the downscaling of model wind speed. *Weather. Forecast.* **19**, 485–495, DOI: 10.1175/1520-0434(2004)019<0485:ACPAFT>2.0.CO;2 (2004). http://dx.doi.org/10.1175/1520-0434(2004)019<0485:ACPAFT>2.0.CO;2.

27. Wieringa, J. An objective exposure correction method for average wind speeds measured at a sheltered location. *Q. J. Royal Meteorol. Soc.* **102**, 241–253, DOI: 10.1002/qj.49710243119 (1976).

28. Wieringa, J. Roughness-dependent geographical interpolation of surface wind speed averages. *Q. J. Royal Meteorol. Soc.* **112**, 867–889, DOI: 10.1002/qj.49711247316 (1986).

29. Rotach, M. W. On the influence of the urban roughness sublayer on turbulence and dispersion. *Atmospheric Environ.* **33**, 4001 – 4008, DOI: http://dx.doi.org/10.1016/S1352-2310(99)00141-7 (1999).

30. Fisher, B. *et al. Meteorology applied to urban air pollution problems – Final Report COST Action 715* (2005).

31. Drew, D., Barlow, J. & Cockerill, T. Estimating the potential yield of small wind turbines in urban areas: A case study for greater london, UK. *J. Wind. Eng. Ind. Aerodyn.* **115**, 104 – 111, DOI: http://dx.doi.org/10.1016/j.jweia.2013.01.007 (2013).

32. Drew, D. R., Barlow, J. F. & Lane, S. E. Observations of wind speed profiles over greater london, uk, using a doppler lidar. *J. Wind. Eng. Ind. Aerodyn.* **121**, 98 – 105, DOI: http://dx.doi.org/10.1016/j.jweia.2013.07.019 (2013).

33. Wood, D. H. Internal Boundary Layer Growth Following a Step Change in Surface Roughness. *Boundary Layer Meteorol.* **22**, 241–244 (1981).

34. Jerolmack, D. J. *et al.* Internal boundary layer model for the evolution of desert dune fields. *Nat. Geosci.* **5**, 206–209 (2012).

35. Savelyev, S. A. & Taylor, P. A. Internal boundary layers: I. height formulae for neutral and diabatic flows. *Boundary-Layer Meteorol.* **115**, 1–25, DOI: 10.1007/s10546-004-2122-z (2005).

36. Bou-Zeid, E., Parlange, M. B. & Meneveau, C. On the Parameterization of Surface Roughness at Regional Scales. *J. Atmospheric Sci.* **64**, 216–227 (2007).

37. Padhra, A. *Estimating the sensitivity of urban surface drag to building morphology.* Ph.D. thesis, University of Reading, UK (2009).

38. Barlow, J. F. Progress in observing and modelling the urban boundary layer. *Urban Clim.* **10, Part 2**, 216 – 240, DOI: http://dx.doi.org/10.1016/j.uclim.2014.03.011 (2014). ICUC8: The 8th International Conference on Urban Climate and the 10th Symposium on the Urban Environment.

39. Mason, P. J. The formation of areally-averaged roughness lengths. *Q. J. Royal Meteorol. Soc.* **114**, 399–420, DOI: 10.1002/qj.49711448007 (1988).

40. Schuepp, P., Leclerc, M., MacPherson, J. & Desjardins, R. Footprint prediction of scalar fluxes from analytical solutions of the diffusion equation. *Boundary-Layer Meteorol.* **50**, 355–373, DOI: 10.1007/BF00120530 (1990).

41. Luhar, A. K., Venkatram, A. & Lee, S.-M. On relationships between urban and rural near-surface meteorology for diffusion applications. *Atmospheric Environ.* **40**, 6541–6553 (2006).

42. Rossby, C.-G. & Montgomery, R. B. The Layer of Frictional Influence in Wind and Ocean Currents. *Pap. Phys. Oceanogr. Meteorol.* **3**, 1–101 (1935).

43. Zilitinkevich, S. & Baklanov, A. Calculation of the height of the stable boundary layer in practical applications. *Boundary-Layer Meteorol.* **105**, 389–409, DOI: 10.1023/A:1020376832738 (2002).

44. Mahrt, L., Andre, J. C. & Heald, R. C. On the Depth of the Nocturnal Boundary Layer. *J. Appl. Meteorol.* **21**, 90–92 (1982).

45. Melgarejo, J. W. & Deardorff, J. W. Revisions to "Stability Functions for the Boundary-Layer Resistance Laws Based Upon Observed Boundary-Layer Heights. *J. Atmospheric Sci.* **32**, 837–839 (1975).

46. Britter, R. E. & Hanna, S. R. Flow and Dispersion in Urban Areas. *Annu. Rev. Fluid Mech.* **35**, 469–496 (2003).

47. Elliott, W. P. The growth of the atmospheric internal boundary layer. *Eos, Transactions Am. Geophys. Union* **39**, 1048–1054, DOI: 10.1029/TR039i006p01048 (1958).

48. Kastner-Klein, P. & Rotach, M. W. Mean flow and turbulence characteristics in an urban roughness sublayer. *Boundary-Layer Meteorol.* **111**, 55–84, DOI: 10.1023/B:BOUN.0000010994.32240.b1 (2004).

49. Hurley, P. The air pollution model (tapm) version 3. part 1. technical description. Tech. Rep. NO. 71., CSIRO Atmospheric Research (2005). http://www.dar.csiro.au/tapm/.

50. Hurley, P. J., Physick, W. L. & Luhar, A. K. Tapm: a practical approach to prognostic meteorological and air pollution modelling. *Environ. Model. & Softw.* **20**, 737 – 752, DOI: http://dx.doi.org/10.1016/j.envsoft.2004.04.006 (2005).

51. Weekes, S. & Tomlin, A. Evaluation of a semi-empirical model for predicting the wind energy resource relevant to small-scale wind turbines. *Renew. Energy* **50**, 280 – 288, DOI: http://dx.doi.org/10.1016/j.renene.2012.06.053 (2013).

52. Geurts, C. C. *Wind-induced pressure fluctuations on building facades.* Ph.D. thesis, Eindhoven University of Technology (1997). DOI: 10.6100/IR495154.

53. Rotach, M. *et al.* BUBBLE - An urban boundary layer meteorology project. *THEORETICAL AND APPLIED CLIMATOLOGY* **81**, 231–261, DOI: 10.1007/s00704-004-0117-9 (2005).

54. Macdonald, R. W., Griffiths, R. F. & Hall, D. J. An improved method for the estimation of surface roughness of obstacle arrays. *Atmospheric Environ.* **32**, 1857–1864 (1998).

55. Oke, T. R. Initial Guidance to Obtain Representative Meteorological Observations at Urban Sites. Tech. Rep. Report nr. 81, World Meteorological Organization (2006).

56. Best, M. *et al.* Small-scale Wind Energy – Technical Report. Tech. Rep., Met Office (2008).

57. Millward-Hopkins, J. T., Tomlin, A. S., Ma, L., Ingham, D. B. & Pourkashanian, M. Aerodynamic parameters of a uk city derived from morphological data. *Boundary-Layer Meteorol.* **146**, 447–468, DOI: 10.1007/s10546-012-9761-2 (2013).
